# High stability resistive switching mechanism of a screen-printed electrode based on BOBZBT_2_ organic pentamer for creatinine detection

**DOI:** 10.1038/s41598-021-03046-9

**Published:** 2021-12-07

**Authors:** Muhammad Asif Ahmad Khushaini, Nur Hidayah Azeman, Ahmad Ghadafi Ismail, Chin-Hoong Teh, Muhammad Mat Salleh, Ahmad Ashrif A. Bakar, Tg Hasnan Tg Abdul Aziz, Ahmad Rifqi Md Zain

**Affiliations:** 1 Institute of Microengineering and Nanoelectronics, Universiti Kebangsaan Malaysia, 43600 Bangi, Malaysia; 2 Department of Electrical, Electronic and Systems Engineering, Universiti Kebangsaan Malaysia, 43600 Bangi, Malaysia; 3 ASASIpintar Program, Pusat GENIUS@Pintar Negara, Universiti Kebangsaan Malaysia, 43600 Bangi, Malaysia

**Keywords:** Chemical physics, Electronics, photonics and device physics, Techniques and instrumentation

## Abstract

The resistive switching (RS) mechanism is resulted from the formation and dissolution of a conductive filament due to the electrochemical redox-reactions and can be identified with a pinched hysteresis loop on the *I*–*V* characteristic curve. In this work, the RS behaviour was demonstrated using a screen-printed electrode (SPE) and was utilized for creatinine sensing application. The working electrode (WE) of the SPE has been modified with a novel small organic molecule, 1,4-bis[2-(5-thiophene-2-yl)-1-benzothiopene]-2,5-dioctyloxybenzene (BOBzBT_2_). Its stability at room temperature and the presence of thiophene monomers were exploited to facilitate the cation transport and thus, affecting the high resistive state (HRS) and low resistive state (LRS) of the electrochemical cell. The sensor works based on the interference imposed by the interaction between the creatinine molecule and the radical cation of BOBzBT_2_ to the conductive filament during the Cyclic Voltammetry (CV) measurement. Different concentrations of BOBzBT_2_ dilution were evaluated using various concentrations of non-clinical creatinine samples to identify the optimised setup of the sensor. Enhanced sensitivity of the sensor was observed at a high concentration of BOBzBT_2_ over creatinine concentration between 0.4 and 1.6 mg dL^−1^—corresponding to the normal range of a healthy individual.

## Introduction

The resistive switching (RS) mechanism is closely related to the electrochemical redox reaction. When an electric field is applied to an electrochemical cell with an active metal electrode, the metal cations are formed from the oxidation process. During this stage, the electrical condition of the cell experiences a high resistance state (HRS). These cations migrate towards- and are reduced at the inert electrode before self-assembled into a conductive filament (CF). This resulted in a low resistance state (LRS) where the resistance in the cell was reduced abruptly. By reversing the voltage bias, the CF is electrochemically dissolved and the cell is reverted to the HRS^1^. The architecture of the RS setup is usually presented by the top-bottom design i.e. the sandwiched structure^2,3^. However, the planar RS setup offers other advantages such as the low-cost fabrication process as well as anti-crosstalk current^4,5^. In planar RS, the CF grows horizontally, avoiding the sneak current path through neighbouring cells^6^.

The condition, on which the CFs are formed and dissolved in the RS cell, greatly depends on the type of electrodes and the electrolyte that facilitate the migration of the cations^7^. For instance, it was reported that the presence of hydrogen and humidity may enhance the metal ion transport thus, impacting the RS response^8,9^. This can be utilized for sensing application as demonstrated in^10^, where, the biomarker for prostate cancer, H_2_O_2_/Sarcosine, was detected through the use of GeO_x_ membrane incorporated in Electrode-Insulator-Semiconductor (EIS) structure. In^11^, with minimal device fabrication procedures, an RS-based ethanol sensor was developed by utilizing a ± 20 V Cyclic Voltammetry (CV) voltage sweep. There, the susceptibility of electronic properties of Zinc oxide to electromagnetic radiation in the UV range was exploited. Meanwhile, Sahu and Jammalamadaka in^12^ proposed an RS device to detect the bovine serum albumin (BSA). The device works based on the change of the cell’s resistance state due to the interference of BSA molecules to the transport properties of the electrolyte, TiO_2_.

This work proposes a screen-printed electrode (SPE) RS-based sensor for creatinine sensing application. Creatinine biomolecule, a waste product from creatinine phosphate breakdown, is an important biomarker for Chronic Kidney Disease (CKD). In an average person, the level of creatinine in serum is between 0.6 and 1.2 mg dL^−1^ (45–140 µM); however, it can increase to 10 folds during the acute phase of CKD and liver disorders^13^. Previously, several research on the electrochemical-based creatinine sensor have utilized the enzymes creatininase (creatinine amidohydrolase, CA), creatinase (creatine amidinohydrolase, CI), and sarcosine oxidase (SO)^14–16^, Despite a high specificity offered, strict storage conditions and the difficulty for enzymes immobilization on electrode matrix resulted from loose binding, denaturation, and loss of enzyme activity are some of the major limitations for enzyme-based sensor^17^. In the present work, the working electrode (WE) of the SPE was modified with a novel small organic molecule (SOM), 1,4-bis[2-(5-thiophene-2-yl)-1-benzothiopene]-2,5-dioctyloxybenzene (BOBzBT_2_) without the addition of the enzymes. SOM has unique physical and chemical properties, low cost and are more environmentally friendly and has seen an increase in interest to be incorporated into the RS devices^18–21^. The branching of dioctyloxy-substituents on phenylene moiety improves the solubility of BOBzBT_2_ apart from preventing self-aggregation among intermolecular structures of the pentamer^22^. Furthermore, the elongation of π-conjugation backbone with additional benzo[b]thiophene rings on BOBzBT_2_ compound accelerates the formation of radical cations thus, increasing the rate of interaction with the creatinine molecules. The interference to the formation and dissolution of the CF, caused by the interaction between BOBzBT_2_ radical cations and creatinine, influencing the resistance state of the system. Consequently, the generated RS pinched hysteresis loop varies and was used to quantify the concentration of the creatinine. The performance of the sensor was investigated using creatinine diluted in deionized water.

## Results and discussion

### Characterization

**Figure 1:**
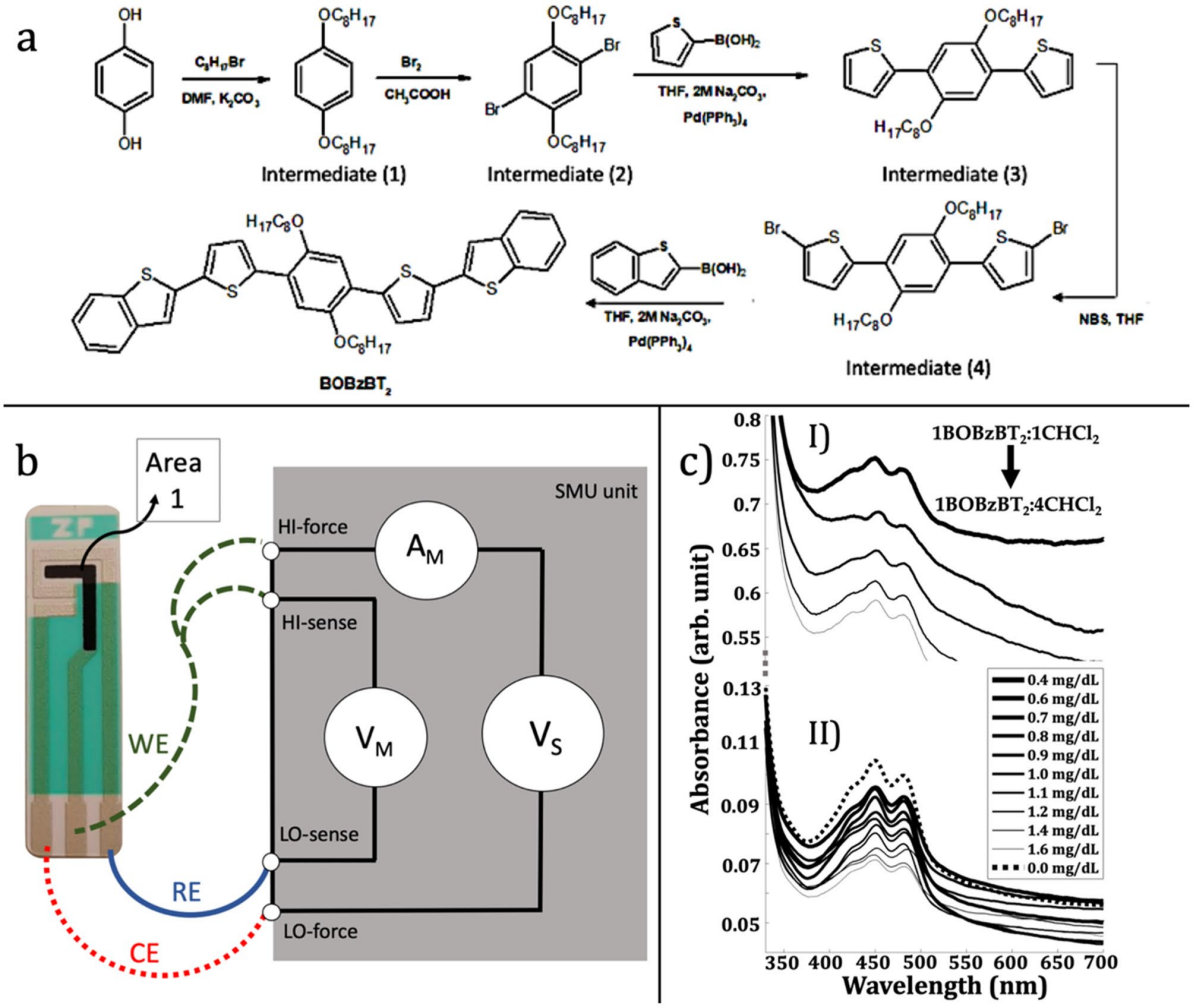
(**a**) The synthetic route of BOBzBT_2_. (**b**) The connection between the electrodes and the SMU instrument where the carbon working electrode (WE) was modified by drop-casting with 2.5µL BOBzBT_2_ dilution (Area 1). The counter electrode (CE) and the reference electrode (RE) are made from Ag/AgCl. (**c**) UV–VIS absorbance spectra for (I) five BOBzBT_2_:CHCl_3_ dilutions and, (II) 3BOBzBT_2_:5CHCl_3_ dilution with ten concentrations of creatinine including the blank solution (0.0 mg dL^−1^).

Figure 1a depicts the synthetic route of the BOBzBT_2_ using 4 intermediate compounds. A schematic of the sensor setup is represented in Fig. 1b, where the cyclic voltammogram (CV) measurement is performed by connecting the SPE to the SourceMeters® unit (SMU). Two attenuated peaks at 450 nm and 481 nm, respectively, were observed from the absorbance spectra of BOBzBT_2_ (Fig. 1c—I) with the respective optical band gap, Eg of 2.94 eV and 2.58 eV. The increase in BOBzBT_2_ concentration in the dilution increased the absorbance spectra since more BOBzBT_2_ molecules are present in the system. The details on the characterization of each intermediate compound and BOBzBT_2_ can be found in our previous works^23,24^. Meanwhile, with the addition of creatinine solution, the absorbance spectra of the 3BOBzBT_2_:5CHCl_3_ dilution were decreased as shown in Fig. 1c—II. This is due to the gradual relaxation between BOBzBT_2_ and the creatinine molecule^25^. Despite that, two BOBzBT_2_ characteristic peaks as obtained previously, were preserved.

### Resistive switching mechanism

**Figure 2:**
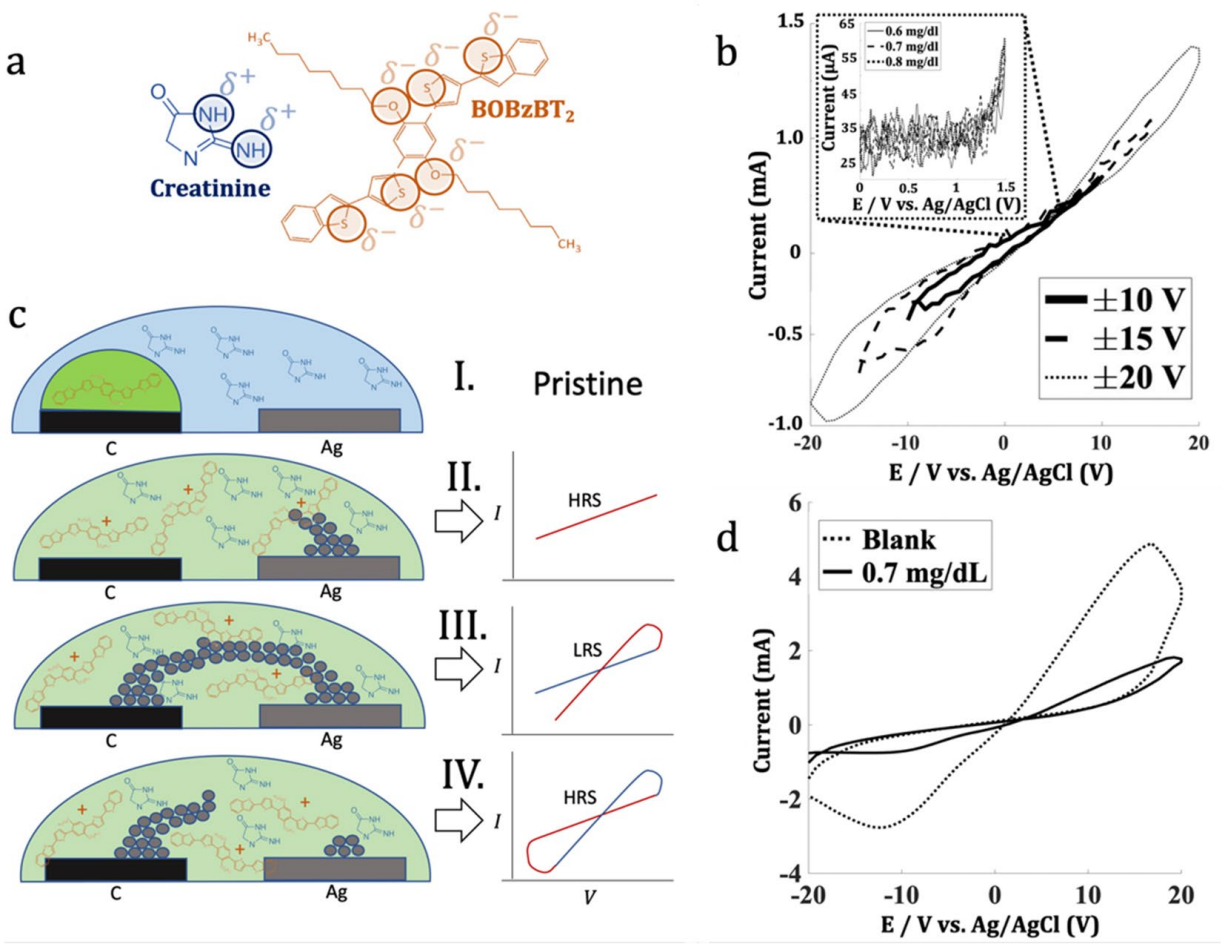
(**a**) The possible interaction sites between BOBzBT_2_ pentamer and creatinine molecule. (**b**) The CV measurement using creatinine concentration of 0.9 mg dL^−1^ at different voltages. The pinched hysteresis loop only is emerged when using ± 20 V. The inset showed attempted CV measurement at 0–1.5 V voltage range on three different concentrations of creatinine. (**c**) The RS mechanism concerning the formation of HRS and LRS, and (**d**) The pinched hysteresis loops obtained from blank solution and 0.7 mg dL^−1^ of creatinine.

The working principle of the sensor is by exploiting the possible polar interaction between creatinine molecule and BOBzBT_2_ pentamer as shown in Fig. 2a. There are two partial negatives, δ^−^ groups in BOBzBT_2_ pentamer, namely at the electron-rich atom such as S at the thiophene unit and O at the alkyloxy side chain. Meanwhile, two partial positive polar groups, δ^+^ in creatinine molecule, were provided by the NH and NH_2_ groups. It is more likely that the polar interaction is formed between S atom at the thiophene or benzothiophene units in BOBzBT_2_ with the NH group in creatinine molecule. This is supported by the fact that the larger red-shift was observed in the N-H···S hydrogen-bonded as compared to the red-shift obtained from the N–H···O hydrogen-bonded as demonstrated in^26^. Moreover, the binding energy of the N-H···S complex was also comparable to that of the indole·benzene complex.

Previously, various voltage ranges, ranging from ± 4 to ± 20 V, were utilized to induce the RS^27–31^. To identify the voltage range suitable for the setup, CV measurements on the sensor modified with the 3BOBzBT_2_:5CHCl_3_ dilution were conducted using different voltage ranges. As shown in the inset of Fig. 2b, the response obtained is highly unstable at a 0–1.5 V voltage range. This is expected due to the simplified electrode preparation resulting in nonuniform BOBzBT_2_ layer. By increasing the voltage ranges to ± 10 V and ± 15 V, the stability of the responses was improved significantly. However, there were no appearances of a pinched hysteresis loop, which is a fingerprint of the RS. Performing the CV measurement with the voltage range of ± 20 V, revealing the pinched hysteresis loop which indicates that the system exhibiting the RS.

The inferred switching mechanism of the electrochemical cell is the formation and dissolution of horizontal CF. During the application of the forward bias voltage, the oxidation at the Ag electrode produces the Ag^+^ cations. The cations were then migrated towards the inert electrode during the state that is referred to as the HRS (as shown in II) in Fig. 2c. At the same time, due to the charge transfer between the BOBzBT_2_ pentamer and the WE, the radical cations of the BOBzBT_2_ were generated. The deposited Ag atoms on the inert electrode self-assemble into Ag CFs (as shown in III) in Fig. 2c resulting in the formation of a conductive route between CE and WE. As consequence, the cell experience an abrupt drop in resistance and the surge of the output current. Following that, the CF is then dissolved bringing the system back to the HRS as represented in (IV) of Fig. 2c. By introducing the creatinine molecules into the electrolyte, the formation and the dissolution of the CF were perturbed due to its interaction with the BOBzBT_2_ radical cations. This is evident by a significant reduction in the LRS peak and the area of the hysteresis loop obtained from the cell with the creatinine solution as compared to the peak obtained from the blank solution (Fig. 2d).

### The optimization of BOBzBT_2_:CHCl_3_ dilutions

**Figure 3:**
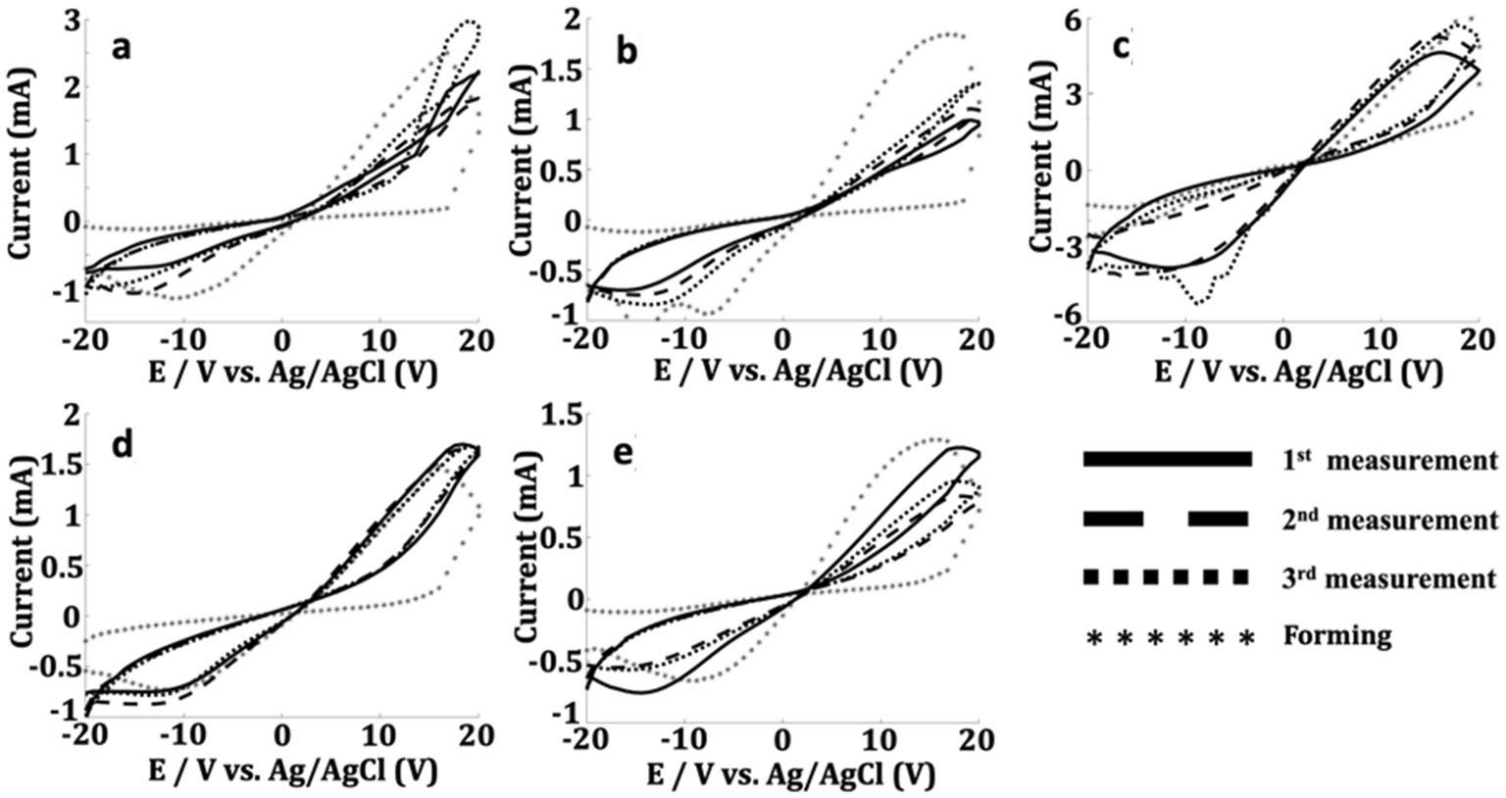
Output currents obtained by performing successive measurements using 0.7 mg dL^−1^ concentration of creatinine for (**a**) 1BOBzBT_2_:4CHCl_3_-sensor, (**b**) 1BOBzBT_2_:3CHCl_3_-sensor, (**c**) 1BOBzBT_2_:2CHCl_3_-sensor, (**d**) 3BOBzBT_2_:5CHCl_3_-sensor and, (**e**) 1BOBzBT_2_:1CHCl_3_-sensor. It is demonstrated that 3BOBzBT_2_:5CHCl_3_-sensor has the highest stability. For the purpose of demonstration, only 3 curves are shown on each figure.

The stability of the sensor modified with different concentrations of BOBzBT_2_ dilution was later evaluated to identify the most optimized setup for creatinine sensing application. By performing the room temperature CV measurements using a single concentration of creatinine (0.7 mg dL^−1^), 5 hysteresis loops were recorded successively after the electroforming process. At low concentrations of BOBzBT_2_ namely 1BOBzBT_2_:4CHCl_3_ and 1BOBzBT_2_:3CHCl_3_, the hysteresis loops noticeably fluctuated in-between the measurements as shown in Fig. 3a, b. The stability between the measurements was seen to have slightly improved at 1BOBzBT_2_:2CHCl_3_ (see Fig. 3c) and with 3BOBzBT_2_:5CHCl_3_ dilution, greater stability between the loops was observed (Fig. 3d). In addition, at all BOBzBT_2_ concentrations, the SET voltage during the electroforming cycle is significantly higher than the SET voltage of the RS cycles. The electroforming process is a crucial step to activate the RS mechanism in the cell, namely by forming the initial path for CF^7^. On the contrary, despite having the highest concentration of BOBzBT_2_, 1BOBzBT_2_:1CHCl_3_-sensor showing moderate stability comparable to medium concentration 1BOBzBT_2_:2CHCl_3_ dilution as shown in Fig. 3e. This may have been resulted from the possible interference with the solvent and surface fouling due to the excessive BOBzBT_2_ pentamer. The precision in-between the measurements was improved significantly in the following order—1BOBzBT_2_:4CHCl_3 _< 1BOBzBT_2_:3CHCl_3 _< 1BOBzBT_2_:1CHCl_3 _< 1BOBzBT_2_:2CHCl_3 _< 3BOBzBT_2_:5CHCl_3_ with the average RSD values of 20.6% < 17.7% < 15.1% < 10.0% < 5.2%, respectively.

**Figure 4:**
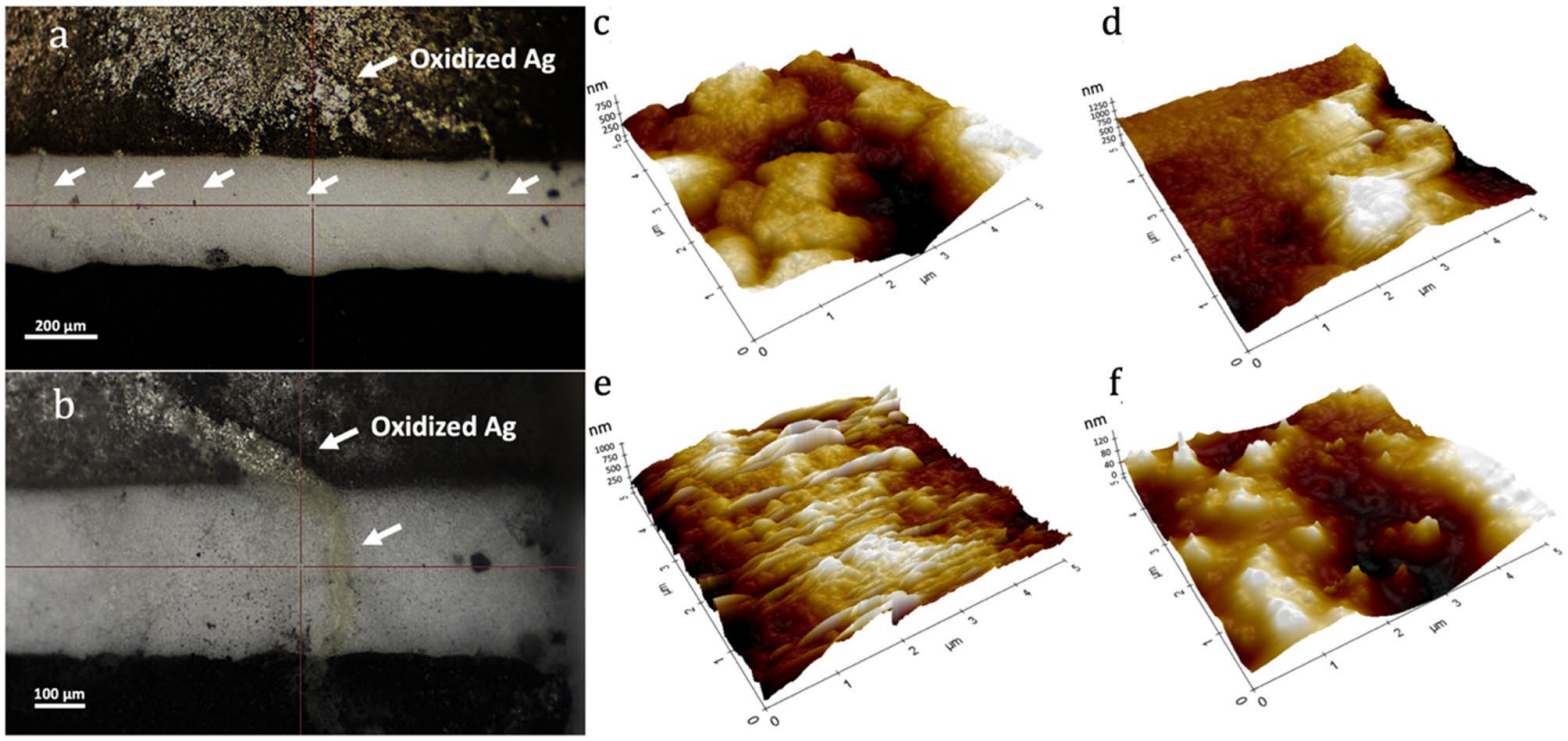
The evidence of CFs, taken after the CV measurement for (**a**) 1BOBzBT_2_:3CHCl_3_-sensor and, (**b**) 3BOBzBT_2_:5CHCl_3_-sensor. AFM-3D images of the bare carbon WE (**c**), after drop casting BOBzBT_2_ dilution onto the WE (**d**), 1BOBzBT_2_:3CHCl_3_-modified WE and 3BOBzBT_2_:3CHCl_3_-modified WE after performing the CV measurements (**e** and **f**, respectively).

The overall stability responses obtained using high voltage cyclic voltammetry throughout the experiment show better film degradation resistance of BOBzBT_2_. It was shown that a large forming voltage is required when the distance between the electrodes is larger^32^. For the commercially available SPE as used in the present work, a relatively large voltage range of ± 20 V is required to induce the RS. However, as the most consequential changes in the structure occur during the forming process, applying such a high voltage can lead to either the soft breakdown or the permanent breakdown^33^. The latter occurs when multiple bridges are produced during the forming process and can result in unstable RS systems. On the contrary, soft breakdown refers to the condition where only a single CF is formed. As shown in Fig. 4a, at a low concentration of BOBzBT_2_ (1BOBzBT_2_:3CHCl_3_ in this example), multiple bridges with thickness ranging from 30 to 50 µm can be observed connecting the active metal electrode and the carbon WE. This may explain the relative instability of the system at lower BOBzBT_2_ concentrations as obtained in Fig. 3a–c. By referring to Fig. 4b, at a higher concentration of BOBzBT_2_ (3BOBzBT_2_:3CHCl3 in this case), a single CF with an average thickness of 70 µm can be seen clearly. The observation implies that more BOBzBT_2_ in the system, provides more control to the formation of the CF, hence contributing to the enhanced stability of the system as in Fig. 3d.

AFM-3D images of the bare carbon WE (Fig. 4c), after drop casting BOBzBT_2_ dilution onto the WE (Fig. 4d), 1BOBzBT_2_:3CHCl_3_-modified WE and 3BOBzBT_2_:3CHCl_3_-modified WE after performing the CV measurements (Fig. 4e, f, respectively) show that morphological change at each process. The surface of the WE showed a significant increase in thickness after the addition of BOBzBT_2_ dilution along with the reduction in the surface roughness parameter, Ra to 26 nm from the average of 44 nm on bare WE. Meanwhile, the value of Ra obtained from 1BOBzBT_2_:3CHCl_3_-modified WE after performing the measurement increased to the average of 120 nm compared to the value before the measurement. Conversely, the surface of 3BOBzBT_2_:3CHCl_3_-modified WE changed to a smooth roughness form with the presence of sparsely located spikes after the measurement. This may be due to coherent electrochemical polymerization onto the WE as a result of the presence of more BOBzBT_2_ pentamers in the cell.

**Figure 5:**
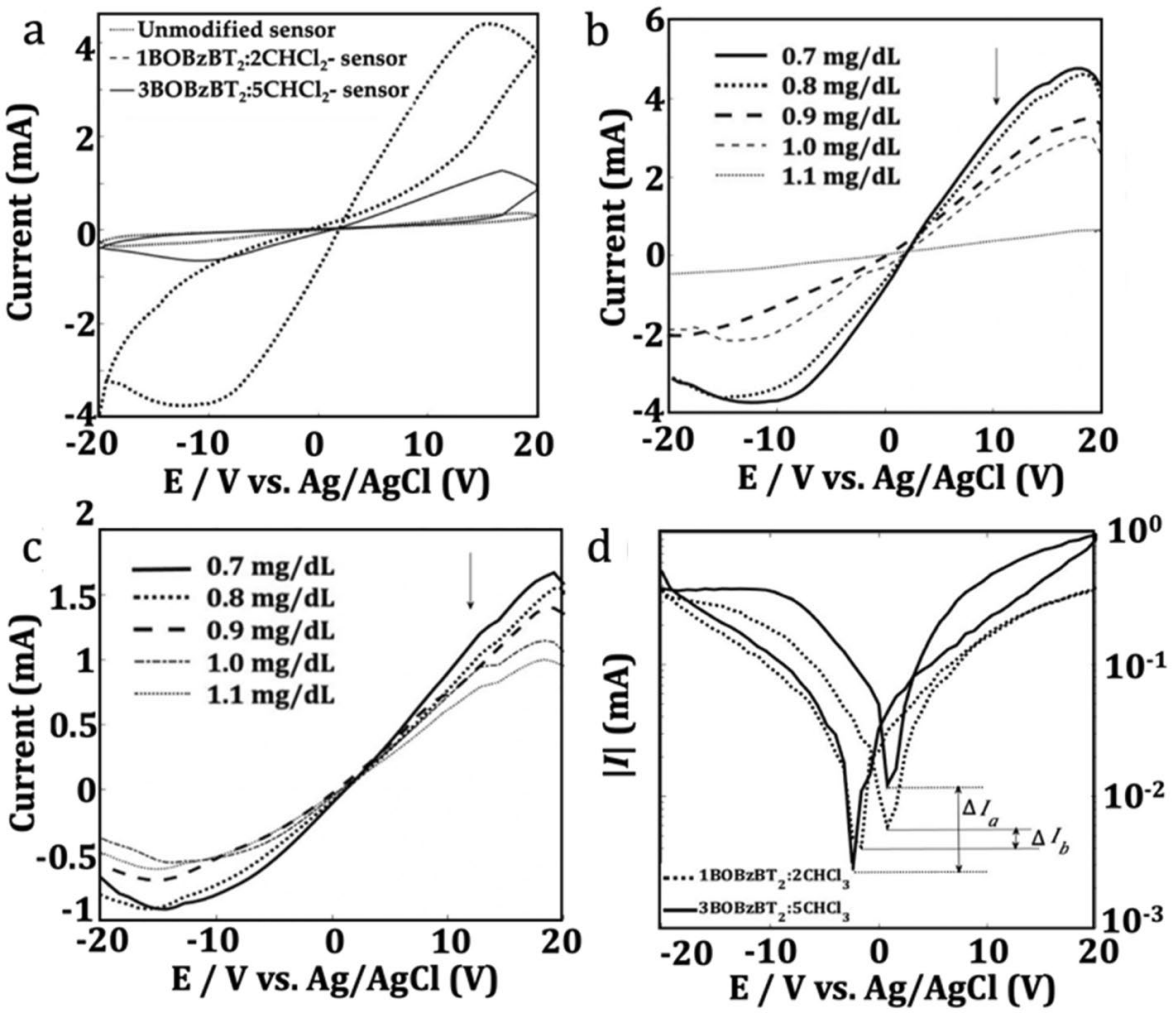
(**a**) Hysteresis loop obtained from performing CV measurement on the unmodified-sensor, 1BOBzBT_2_:2CHCl_3_-sensor and 3BOBzBT_2_:5CHCl_3_-sensor, respectively, using creatinine concentration of 0.7 mg dL^−1^. *I*–*V* curves using four creatinine concentrations for (**b**) 1BOBzBT_2_:2CHCl_3_-sensor and (**c**) 3BOBzBT_2_:5CHCl_3_-sensor, where only the reverse voltage output is shown. (**d**) *I*–*V* curves with log-scale vertical axis using creatinine concentration of 0.7 mg dL^−1^.

The most stable 3BOBzBT_2_:5CHCl_3_-sensor is then compared with the unmodified-sensor and the middle concentration, 1BOBzBT_2_:2CHCl_3_-sensor using 0.7 mg dL^−1^ creatinine solution. Hysteresis loops obtained from 1BOBzBT_2_:2CHCl_3_-sensor and 3BOBzBT_2_:5CHCl_3_-sensor were noticeably larger than the unmodified-sensor as shown in Fig. 5a, suggesting that the RS yield is improved by the presence of the BOBzBT_2_ pentamer. The interaction between BOBzBT_2_ radical cations and the creatinine molecules impedes the mobility of Ag^+^ cations to form the CF. On the other hand, different concentrations of BOBzBT_2_, i.e. 1BOBzBT_2_:2CHCl_3_ and 3BOBzBT_2_:5CHCl_3_, generate a dissimilar concentration of BOBzBT_2_ radical cations; hence, producing different resistance states in the cell. This explained the larger loop obtained from 1BOBzBT_2_:2CHCl_3_-sensor compared to the loop obtained from 3BOBzBT_2_:5CHCl_3_-sensor. Despite that, the output currents obtained from 1BOBzBT_2_:2CHCl_3_ reduced inconsistently as the concentration of creatinine increased (Fig. 5b). In comparison, the reduction of peak current in the 3BOBzBT_2_:5CHCl_3_-sensor varies consistently according to the change of creatinine concentration (Fig. 5c). The occurrence indicates that the stability in the 3BOBzBT_2_:5CHCl_3_-sensor is better compared to the 1BOBzBT_2_:2CHCl_3_-sensor.

Taking the absolute values of output current and observing the logarithmic dependence in respect to the applied voltage, reveals a voltage gap between current minima obtained from forward and reverse loops. By referring to Fig. 5d, as the concentration of BOBzBT_2_ increased from 1BOBzBT_2_:2CHCl3 to 3BOBzBT_2_:5CHCl3, the gap was reduced. This is expected since more radical cations of BOBzBT_2_ are available for the interaction with creatinine molecules hence, restricting the activities of residual charges. Besides that, a larger variation of current minima, $\Delta I _{a}$ was observed from the 3BOBzBT_2_:5CHCl_3_-sensor compared to the variation, $\Delta I _{b}$ obtained from 1BOBzBT_2_:2CHCl_3_-sensor. The variation may indicate an increase in analyte uptake as the consequence of increasing the BOBzBT_2_ concentration in the system^34^. However, one should be aware that this may also be contributed by parasitic resistance present in the system.

### Response time and the dynamics of the system

**Figure 6:**
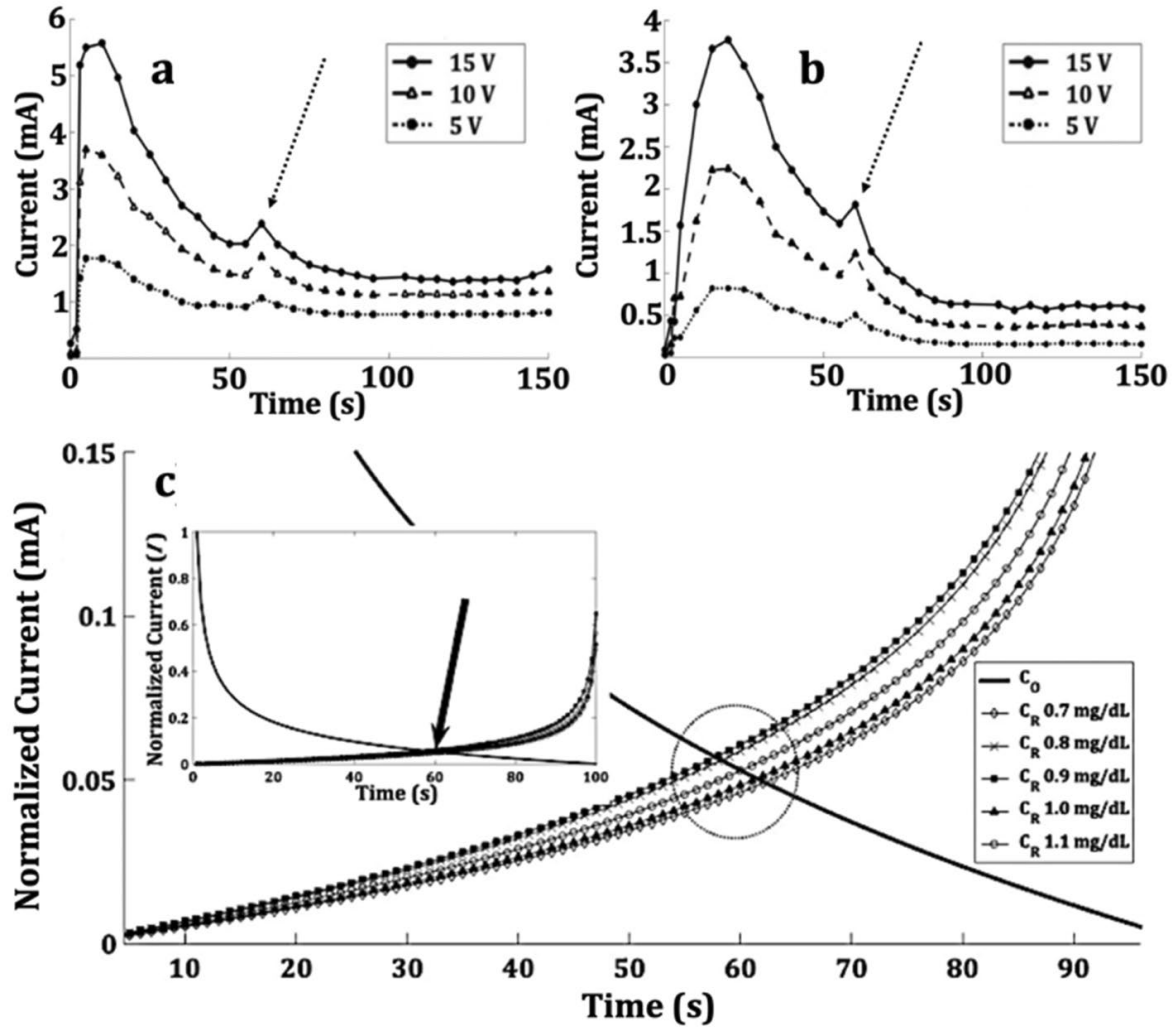
Response time of 3BOBzBT_2_:5CHCl_3_-sensor at 15 V, 10 V and 5 V for (**a**) 0.7 mg dL^−1^ creatinine concentration, (**b**) 1.2 mg dL^−1^ creatinine concentration. (**c**) Graph of normalised current obtained by solving Eq. (1) represented by smooth curve and Eq. (2) represented by marked curves for *t* $\to \infty$. The arrow indicates that the intersection between ${C}_{O}$ and ${C}_{R}$ which is coincided with a spike in (**a**) and (**b**).

One of the characteristics of a sensor is the time it takes to reach the steady-state response. The response time curve can be used as a reference time frame at which the measurement of the analyte can be made. To observe the response time of 3BOBzBT_2_:5CHCl_3_-sensor, consecutive CV measurements were conducted with 5 s time intervals in-between the measurement. The current output values at 5 V, 10 V, and 15 V were then extracted and plotted with respect to time. As a result of electroforming process, the output current demonstrates a sharp increase and reached a peak at *t* = 10 s for creatinine concentration of 0.7 mg dL^−1^ (Fig. 6a). Meanwhile, by using a creatinine concentration of 1.2 mg dL^−1^ i.e. with the increase of creatinine molecules in the system, the steady-state response was achieved at a much later time i.e. at *t* = 20 s (Fig. 6b). This suggests the electroforming process was interrupted with more creatinine-BOBzBT_2_ radical cations interactions.

A noticeable spike of output was observed at approximately *t* = 60 *s* for 3BOBzBT_2_:5CHCl_3_-sensor in 0.7 mg dL^−1^ and 1.2 mg dL^−1^ of creatinine concentrations (dashed arrows in Fig. 6a, b). Interestingly, it was preserved throughout all tested voltages. To investigate this occurrence, the following Cottrell equations were employed as below;

1 $$ i_{ + } \left( t \right) = \frac{{nFAD_{ + } C_{O} }}{{\left( {\pi D_{ + } t} \right)^{1/2} }} $$

2 $$ i_{ - } \left( t \right) = \frac{{nFAD_{ - } C_{R} }}{{\left( {\pi D_{ + } t} \right)^{1/2} }} $$

where $$C_{O} $$ and $$C_{R} $$ are the concentration of oxidised (*O*) and reduced (*R*) forms of electroactive species, respectively. Meanwhile, $$D_{ + }$$ and $$D_{ - }$$ are the diffusion coefficients during the forward bias, calculated using maximum current, $$i_{p, + }$$ and the reverse bias, calculated using modulus of minimum current, $$i_{p, - }$$, respectively. The process of estimating the value of $$D_{ + }$$ and $$D_{ - }$$ can be found in the Supplementary Information. By solving both equations for *t* $$\to \infty$$ and plotting the results against the normalised output current for four different concentrations of creatinine, it was demonstrated that there is an intersection between $$C_{O} $$ and $$C_{R} $$ in the range of 58 s–62 s, indicating an equal presence of *O* and *R* in the system (Fig. 6c).

### Sensor performance for quantifying creatinine concentration

**Figure 7:**
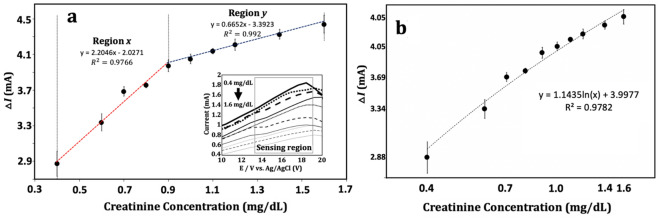
(**a**) Calibration curve (*N* = 5) for 3BOBzBT_2_:5CHCl_3_-sensor shows two linear regression regions namely region $x$, 0.4 mg dL^−1^ $\le x\le$ 0.9 mg dL^−1^ and region $y$, 0.9 mg dL^−1^ $\le y\le$ 1.6 mg dL^−1^ with correlation coefficients R^2^ of 0.977 and 0.992, respectively. (**b**) The log–log calibration curve (*N* = 5) with correlation coefficients R^2^ of 0.978.

To investigate the performance of 3BOBzBT_2_:5CHCl_3_-sensor, a calibration curve was established by taking the output current at voltage value of 15 V measured at each creatinine concentration. This value is then subtracted by the output measured from performing the measurement using a blank solution (0.0 mg dL^−1^) to obtained, $\Delta I$. By referring to Fig. 7a, the working range of the 3BOBzBT_2_:5CHCl_3_-sensor was 0.4–1.6 mg dL^−1^ of creatinine. Despite the subtracted output current, $\Delta I$ rises steadily with a concentration in this range, two linear regressions can be drawn from the curve namely, one for the low concentration range of 0.4 mg dL^−1^ $\le x\le$ 0.9 mg dL^−1^ (region $x$ in Fig. 7a) and one for the high concentration range of 0.9 mg dL^−1^ $\le y\le$ 1.6 mg dL^−1^ (region $y$ in Fig. 7a). These regions *x* and *y* were fitted to the data with correlation coefficients R^2^ of 0.977 and 0.992, respectively. Despite larger R^2^, the sensitivity, taken from the slope of the linear regression line; for the region $y$ shows a 70% reduction at 0.665 A dL g^−1^ as compared to 2.204 A dL g^−1^ obtained from the region $x$. The finding is similar to the observation reported in^35^ which suggested that two linear ranges with lower sensitivity at higher concentration regions may have been due to the accumulation of the analyte on the modified electrode.

The value of the limit of detection (LOD) region $x$ and $y$ were determined at 0.20 mg dL^−1^ and 0.18 mg dL^−1^, respectively while the limit of quantification (LOQ), was calculated at 0.63 mg dL^−1^ for region $x$ and 0.55 mg dL^−1^ region $y$. Both the LOD and LOQ were calculated based on the ICH Topic Q2 (R1) Validation of Analytical Procedures: Text and Methodology, namely clauses 6 and 7, respectively. Meanwhile, by transforming both axes of the calibration curve (Fig. 7b) to the logarithmic scale, regions $x$ and $y$ can be effectively combined into a single pseudo-linear regression line with a relatively high value of R^2^ = 0.978.

**Figure 8:**
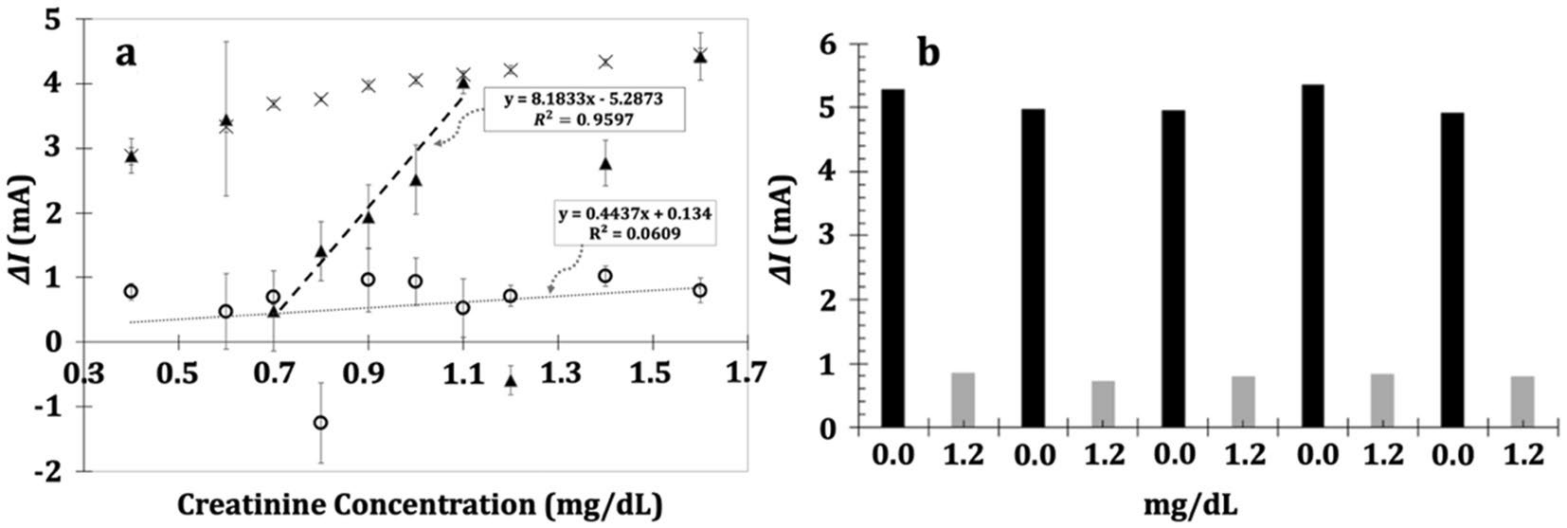
(**a**) The calibration curves obtained from the 3BOBzBT_2_:5CHCl_3_-sensor (**X**), 1BOBzBT_2_:2CHCl_3_-sensor (black filled triangle), and the unmodified-sensor (open circle). The thick dashed line represents the linear regression for 1BOBzBT_2_:2CHCl_3_-sensor while the thin dotted line represents the attempted linear regression for the unmodified-sensor. (**b**) Reproducibility bar obtained by recording in repetition, the peak output currents of blank solution (0.0 mg dL^−1^; black bar) and the creatinine concentration of 1.2 mg dL^−1^ (grey bar).

To further demonstrate the improvement made by the BOBzBT_2_ on the sensor performance, the calibration curves obtained from the unmodified-sensor, 1BOBzBT_2_:2CHCl_3_-sensor, and 3BOBzBT_2_:5CHCl_3_-sensor were compared (Fig. 8a). No linear operating range can be established from the unmodified-sensor due to the fluctuation of the output currents for the concentrations. This further suggests that, in the absence of BOBzBT_2_ in the cell, the CF was not modulated, resulting in the incoherence formation and dissolution process of the CF. Moreover, as previously demonstrated in Fig. 5a, the narrower loop obtained from the unmodified sensor indicates that the cell response approaches that of the ohmic-like behaviour. The addition of BOBzBT_2_ enhanced the retention of the CF similar to the observation reported in^33^, where the addition of defective graphene into Ag/SiO_2_/Pt cell led to the highly stable and robust CF during the HRS phase. On the other hand, it was observed that the sensor with 1BOBzBT_2_:2CHCl3 dilution exhibits a limited detection range of 0.7 mg dL^−1^ and 1.1 mg dL^−1^ compared to the 3BOBzBT_2_:5CHCl_3_-sensor. This may have been caused by branching and the formation of multiple CFs as discussed in the previous section, predominantly at the lower (1.1 mg dL^−1^) concentrations of creatinine. Modest performance for the sensor with 1BOBzBT_2_:2CHCl_3_ dilution was also obtained with larger LOD and LOQ of 0.36 mg dL^−1^ and 1.08 mg dL^−1^, respectively.

The analysis of variance (ANOVA) was later conducted to analyze the statistical significance of the variance within the group as represented by the error bar and between the groups, i.e. between the BOBzBT_2_- and unmodified-sensors. The null hypothesis is set to no difference between all three sensors. The Sum of Squares Within Groups (SSW), Sum of Squares between Group (SSG), and Total Sum of Squares (SST) for the sensors were determined to obtain the value of F-ratio at each creatinine concentration. Using the confidence level of 95% and the numerator and the denominator degrees of freedom are 2 and 9, respectively, the critical value of F(2,9) is given at 4.256. It was calculated that the value of F is larger than the critical value at all tested creatinine concentrations as listed in Table S.1 in the Supplementary Information. A larger calculated F value as compared to the critical value indicates that the variation among group means is significant. This is thus concluded that the null hypothesis can be rejected hence the difference between 3BOBzBT_2_:5CHCl_3_-sensor, 1BOBzBT_2_:2CHCl_3_-sensor and unmodified-sensor are statistically significant.

The selectivity of the modified-SPE was evaluated by conducting CV measurements using different concentrations of human serum albumin (HSA) solution. The HSA was chosen since it has the net negative charge^36^ i.e., a direct opposition from the inferred interaction between the partial negative group in the BOBzBT_2_ and the partial positive group in the creatinine molecule. As demonstrated in Fig. S1 in the Supplementary Information, only Ohmic-like responses were recorded at different HSA concentrations, except for 0.8 mg dL^−1^ of HSA. Despite the visible pinched hysteresis loop at this concentration, the area of the loop is extremely narrow compared to the loop obtained from using the creatinine solutions. This implies that the interaction between HSA molecules and the BOBzBT_2_ radical cations is inadequate to modulate the resistance state of the cell; thus, the efficiency of the cell to induce the resistive switching is low. Moreover, no working range could be established (inset of Fig. S1 in the Supplementary Information) suggesting the selectivity of modified-SPE towards the creatinine molecules over the HSA molecules.

The utilization of a large voltage range to induce the RS mechanism comes with the expense of deteriorating the electrodes. As a result, the sensor strip is a single use which can be demonstrated by performing repeatability testing. The test was conducted by rinsing the strip with DI water after each measurement. The rinsed strip is then left dried before being drop-casted with 3BOBzBT_2_:5CHCl_3_ dilution. As shown in Fig. S2 in the Supplementary Information, after the measurement using the fresh strip, there were no usable signals that can be recorded from the subsequent measurements. The reproducibility of the sensor was later investigated by conducting CV measurement for a blank solution followed by a measurement using 1.2 mg dL^−1^ creatinine concentration. The step was repeated five times and the results have demonstrated that the sensor response is consistent throughout the cycle, indicating its high reproducibility (Fig. 8b). On the other hand, the comparison between the LOD and the detection range of 1BOBzBT_2_:2CHCl_3_- and 3BOBzBT_2_:5CHCl_3_- sensors with other methods proposed in the literature are summarized in Table S.2 in the Supplementary Information. Despite the low voltage CV offered in those methods, the variation of the output current is minuscule, namely at the scale of ~µA as compared to the ~mA variation offered by the present work. This eliminates the necessity of having a sensitive output detector. Furthermore, the simplicity of the sensor preparation, namely, through drop-casting the BOBzBT_2_:CHCl_3_ dilution onto the WE, may open a possibility of an alternative quantification technique of creatinine concentration.

## Conclusions

We demonstrated a low-cost, RS-based creatinine sensor using disposable screen-printed electrode (SPE). The working electrode (WE) of the SPE was modified with a small organic molecule, 1,4-bis[2-(5-thiophene-2-yl)-1-benzothiopene]-2,5-dioctyloxybenzene (BOBzBT_2_). The polar interaction occurred in the electrolyte, namely, between the radical cations of BOBzBT_2_ pentamer and creatinine molecules influencing the resistance state of the cell. As a result, the RS responses i.e. the pinched hysteresis loops varied with respect to the creatinine concentrations. Accordingly, the quantification of creatinine concentration was carried out based on the reduction of output current as creatinine concentration increased. BOBzBT_2_ dilution with the ratio of 3BOBzBT_2_:5CHCl_3_ has improved the overall performance of the sensor with the sensitivity of 2.204 A dL g^−1^ and 0.665 A dL g^−1^ for region $x$ and region $y$, respectively. The sensor responded linearly over the concentration range 0.4 mg dL^−1^ to 1.6 mg dL^−1^, corresponding to the creatinine level in serum for healthy individuals. Meanwhile, the limit of detection (LOD) of the sensor is as low as 0.18 mg dL^−1^. Minimal fabrication procedures offered by the setup along with a good sensing performance may pave a way for the development of a RS-based SPE sensor for quantifying creatinine concentration. Moreover, by functionalizing BOBzBT_2_ with a particular receptor, the selectivity and the specificity of the sensor can be further improved hence enabling the real sample measurement.

## Methods

### Materials

Materials were purchased from commercial sources (Sigma Aldrich, Acros Organic, Alfa Aesar and Merck Millipore). 1-Bromooctane (99%), hydroquinone (99%), bromine (99%), 2-thiophene boronic acid (98%), benzo[b]thien-2-ylboronic acid (99%), tetrakis(triphenylphosphine) palladium(0) [Pd(PPh_3_)_4_] (99%) and N-bromosuccinimide (NBS) (99%) were used as received. Reagent grade tetrahydrofuran (THF) and dimethylformamide (DMF) was distilled, respectively, from sodium/benzophenone and calcium hydride (CaH) before use, according to the work reporting elsewhere^37^.

### Preparation of BOBzBT_2_

The BOBzBT_2_ was synthesized through 4 intermediate compounds namely 1,4-bis(octyloxy)benzene (1),2,5-dibromo-1,4-bis(octyloxy)benzene (2), 1,4-bis(thiophen-2-yl)-2,5-dioctyloxybenzene (3) and 1,4-bis(5-bromo-thiophen-2-yl)-2,5-dioctyloxybenzene (4) as shown in Fig. 1a. Williamson etherification reaction was used to prepare the intermediate (1) followed by the use of bromination reaction using bromine in acetic acid glacial solution to obtain the intermediate (2). The procedure is similar to that used in^38^. The Suzuki cross-coupling reaction was later performed to synthesize intermediate (3) before undergoing bromination reaction using NBS in THF solution to obtain the intermediate (4). Following the procedures of in^24^, the thiophene boronic acid on intermediate (4) was then replaced with benzo[b]thien-2-ylboronic acid to obtain 1,4-bis[2-(5-thiophen-2-yl)-1-benzothiophene]-2,5-dioctyloxybenzene (BOBzBT_2_) pentamer. Five dilutions with different BOBzBT_2_:CHCl_3_ ratios (mg:ml) were prepared namely, 1BOBzBT_2_:4CHCl_3_, 1BOBzBT_2_:3CHCl_3_, 1BOBzBT_2_:2CHCl_3_, 3BOBzBT_2_:5CHCl_3_, 1BOBzBT_2_:1CHCl_3_ arranged in ascending order according to the increase of BOBzBT_2_ concentration. The details regarding the synthesis procedure of BOBzBT_2_, as well as the characterization and the spectra, can be found in Supplementary Information.

### Preparation of creatinine stock solution

Creatinine stock solution (5 mg dL^−1^) was prepared by dissolving 12.5 mg of creatinine with deionised water in a 250 ml (2.5 dL) volumetric flask. A series of creatinine dilutions was then prepared from a stock solution ranging from 0.4 to 1.6 mg dL^−1^ and used as a standard working solution. The range of concentration was chosen based on the standard concentration level of creatinine in serum of healthy individuals^13^.

### UV–VIS spectrophotometry

A Hitachi U-3900H Spectrophotometer was used to measure the absorption spectra for each ratio of BOBzBT_2_:CHCl_3_ dilution and 3BOBzBT_2_:5CHCl_3_ dilution with the addition of different concentrations of creatinine. The sample was prepared by dropping 2.5µL of BOBzBT_2_:CHCl_3_ dilution onto the glass slide and was left for 5 min drying at room temperature. Meanwhile, a single concentration of dilution, namely 3BOBzBT_2_:5CHCl_3_ was randomly selected to observed the UV-VIS absorbance spectra in the presence of creatinine solutions. A droplet of creatinine solution at ten different concentrations was added on top of 10 glass slides with each containing 2.5µL of 3BOBzBT_2_:5CHCl_3_ that was left dried for 5 min. The spectrophotometry scanning was performed at a 300nm/min scan rate with a 300–700nm wavelength range.

### Electrochemical measurement

The sensor used a three-electrode screen-printed strip (Zimmer Peacok; "SPE") with a carbon working electrode (WE) (0.038cm^−2^ of area) and Ag/AgCl electrode for both reference (RE) and counter electrodes (CE) (Fig. 1). Amperometric measurements were performed using a Keithley 2400 series 200 V, 1A, 20 W SMU. The potential sweeping is applied by the voltage source (V_S_) between the WE and the CE. The potential across the RE and WE are measured with the voltmeter, V_m_ and to maintain the intended potential at WE with respect to RE, V_s_ is adjusted accordingly. Subsequently, the resulting current flowing to or from WE is measured with the ammeter (A_M_).

### Electrode preparation

Before the measurement, 2.5 µL of BOBzBT_2_ dilution was drop-casted on WE (Area 1 in Fig. 1) and left for several minutes to dry at room temperature. Next, a droplet of creatinine solution was added in the vicinity of Area 1, which covered all three electrodes and left for another few minutes, allowing the chemical relaxation in the system to occur. The cyclic voltammetry measurement was performed, and the obtained output current was recorded. The procedure is repeated for all five ratios of BOBzBT_2_: CHCl_3_ dilution, at which ten different concentrations of creatinine solutions were used to identify the most optimised dilution for the sensor.

## Supplementary Information

Supplementary Information 1.
