# Supplementary material for: High stability resistive switching mechanism of a screen-printed electrode based on BOBZBT2 organic pentamer for creatinine detection
Source: Sci Rep. 2021 Dec 7;11:23519. doi: 10.1038/s41598-021-03046-9 (PMC8651797; doi:10.1038/s41598-021-03046-9)
Supplement: Supplementary file 1 — Supplementary Information 1. [file 41598_2021_3046_MOESM1_ESM.docx]

**Supplementary Information**

**S.1. Switching kinetics and the estimation of diffusion coefficients**

At all of the measurements conducted on the modified sensors, it is observed that the magnitude of the maximum current output at the positive voltage range (0 – 20 V) is larger compared to the magnitude of the minimum current output obtained from the negative voltage range (-20 – 0 V). This can be interpreted by the difference in intrinsic resistance after the formation and after the rupture of the conductive filament. The diffusion coefficient can be estimated by using the Randles–Sevcik equations [30,31];

|  | $D_{+}=\frac{i_{p,+}}{\left( 2.99 \times{10}^{5} \right)nAC_{B}v^{1/2}}$ | (1) |
| --- | --- | --- |

|  | $D_{-}=\frac{i_{p,-}}{\left( 2.99 \times{10}^{5} \right)nAC_{B}v^{1/2}}$ | (2) |
| --- | --- | --- |

Where $n$, $A$, $C_{B}$ and $v$ is the number of electrons involve in the process, area of the WE, the bulk concentration of the creatinine solution and scan rate, respectively. $n$ is set at 1 while the scan rate use in the study is 8 V s^-1^ . Here, $D_{+}$ and $D_{-}$ are the diffusivity during the forward bias, calculated using maximum current, $i_{p,+}$ and the reverse bias, calculated using modulus of minimum current, $i_{p,-}$ , respectively. The application of equation (1) and (2) is under the assumption that the creatinine molecule is the dominating ion and was chosen instead of Ag^+^, considering changes to the output current when varying the concentration of creatinine. At creatinine concentration of 0.7, 0.8 0.9, 1.0, 1.1 mg dL^-1^, the decrease of $D_{-}$in respect to $D_{+}$ , in term of the percentage, for 3BOBzBT_2_:5CHCl_3_-sensor are 76%, 61%, 58%, 73% and 68% respectively. On the other hand, 28%, 29%, 59%, 48%, and 37% decrease of $D_{-}$ for the same creatinine concentrations were observed for 1BOBzBT_2_:2CHCl_3_-sensor, respectively. A larger $D_{-}$ reduction obtained from 3BOBzBT_2_:5CHCl_3_-sensor compared to 1BOBzBT_2_:2CHCl_3_-sensor suggests that there was an increase in efficiency of BOBzBT_2_-creatinine interaction. Besides, a significant reduction of $D_{-}$and $i_{p,-}$in 3BOBzBT_2_:5CHCl_3_- sensor, attributed by faster perturbation of creatinine molecule onto BOBzBT_2_ radical cations similar to the reaction between radical cations of drug chlorpromazine with water [33].

1. Tappertzhofen, S., Mündelein, H., Valov, I., & Waser, R. Nanoionic transport and electrochemical reactions in resistively switching silicon dioxide. *Nanoscale*, *4*(10), 3040. <https://doi.org/10.1039/c2nr30413a> (2012).
2. Messerschmitt, F., Kubicek, M., Schweiger, S., & Rupp, J. L. M. Memristor kinetics and diffusion characteristics for mixed anionic-electronic srtio _3-δ_ bits: The memristor-based cottrell analysis connecting material to device performance. *Advanced Functional Materials*, *24*(47), 7448–7460. <https://doi.org/10.1002/adfm.201402286> (2014).
3. Arimi, A., Dillert, R., Dräger, G., & Bahnemann, D. W. Light-induced reactions of chlorpromazine in the presence of a heterogeneous photocatalyst: Formation of a long-lasting sulfoxide. *Catalysts*, *9*(7), 627. <https://doi.org/10.3390/catal9070627> (2019).

**S.2. Selectivity and repeatability of the 3BOBzBT_2_:5CHCl_3_-sensor.**


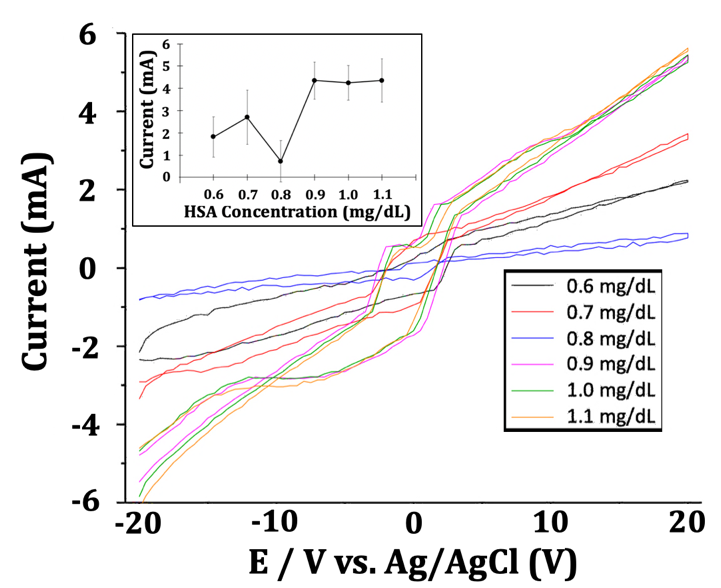


**Figure S1.** The CV measurement for the HSA with concentration range of 0.6 – 1.1 mg dL^-1^ which implies that the interaction between HSA molecules and the BOBzBT_2_ radical cations is inadequate to modulate the resistance state of the cell. Inset shows that the output at 15 V taken from each measurement reveals no linear relationship with the concentration of HSA.


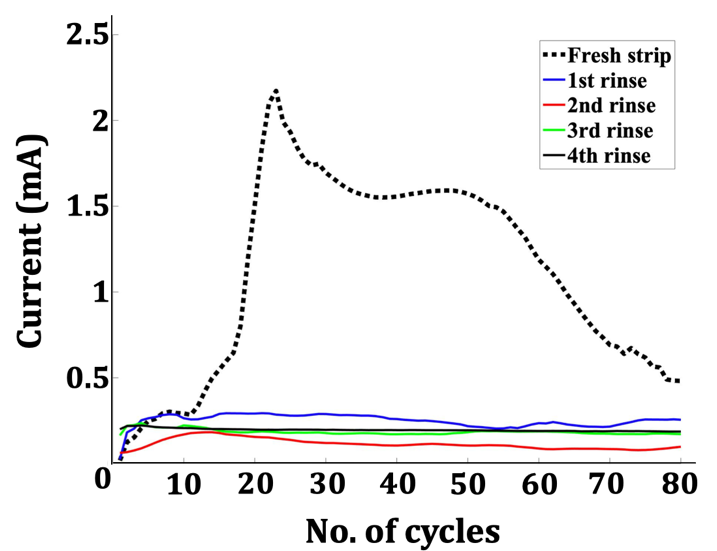


**Figure S2.** The repeatability test of the 3BOBzBT_2_:5CHCl_3_-sensor. The output currents extracted at 15 V for creatinine concentration of 1.0 mg dL^-1^ at each cycle demonstrate that the sensor strip is a single use, i.e. only the fresh strip produced a useable signal.

**S.3. Sensor Performance for Quantifying Creatinine Concentration**

|  | **Creatinine concentration (mg/dL)** | | | | | | | | | |
| --- | --- | --- | --- | --- | --- | --- | --- | --- | --- | --- |
|  | **0.4** | **0.6** | **0.7** | **0.8** | **0.9** | **1.0** | **1.1** | **1.2** | **1.4** | **1.6** |
| F(2,9) | 20.4 | 392.2 | 104.7 | 92.4 | 128.0 | 912.2 | 154.3 | 5.9 | 126.3 | 462.8 |

**Table S.1.** The value of ratio of the between group variance to the within group variance, F(2,9) at at each creatinine concentration.

| **Setup** | **Electrode preparation** | **Sensitivity** | **LOD**  **(mg/dL)** | **Range (mg/dL)** | **Applied potential** | **Output current** | **Ref** |
| --- | --- | --- | --- | --- | --- | --- | --- |
| Enzymatic  Amperometric | Colloidal gold into the MWCNT; addition HRP and Teflon; Drop of mixture of CI, CA and SO enzymes; Stored at 4°C. | 1.91  (µA /mmL^-1^) | 0.001 | 0.034 to 11.31 | -0.3-0.9  (V) | -25-25  (µA) | [1] |
| Enzymatic  Amperometric | Mixing of graphite powder with Fe_3_O_4_ nanoparticles; Mixing with enzyme solution. | 1.82  (µM^-1^) | 0.002 | 0.002 - 0.043 | -0.4-0.8  (V) | -1.5-1.5  (µA) | [2] |
| Enzymatic  Amperometric | Dissolving FcMeOH in carbon ink; deposited onto PET; printing of the mixture of enzymes CI, CA and SO. | 0.38  (A/M) | 0.027 | 0.056 to 11.3 | -0.2-0.6  (V) | -2-2  (µA) | [3] |
| Non-enzymatic  Amperometric | Hydrothermal reaction of GO with dopamine HCl; polymerisation of PDA; Electrodeposition of Cu. | 1.59  (µA/mM) | 0.00002 | 0.0001 to 1.13 | -0.2-0.8  (V) | -0.6-0.6  (mA) | [4] |
| Non-enzymatic  RS-based | Drop cast 2.5µL of 1BOBzBT_2_:2CHCl_3_ dilution; left dried at room temperature. | 8.18  (A dL g^-1^) | 0.36 | 0.7 – 1.1 | -20-20  (V) | -4-6  (mA) | Present work |
| Non-enzymatic  RS-based | Drop cast 2.5µL of 3BOBzBT_2_:5CHCl_3_ dilutio; left dried at room temperature. | 2.20 (region $x$) 0.67 (region $y$)  (A dL g^-1^) | 0.2 (region $x$) /  0.18 (region $y$) | 0.4 – 1.6 | -20-20  (V) | -1-2  (mA) | Present work |

**Table S.2.** The comparison of present work with the selected literature demonstrates the simplification of the BOBzBT_2_ sensor.

1. Kaçar, C., Erden, P. E., Pekyardimci, Ş., & Kiliç, E., An Fe _3_ O _4_ -nanoparticles-based amperometric biosensor for creatine determination. Artificial Cells, Nanomedicine, and Biotechnology (2013) 41(1), 2–7. <https://doi.org/10.3109/10731199.2012.712044> (2012).
2. Gao, X., Gui, R., Guo, H., Wang, Z., & Liu, Q., Creatinine-induced specific signal responses and enzymeless ratiometric electrochemical detection based on copper nanoparticles electrodeposited on reduced graphene oxide-based hybrids. Sensors and Actuators B: Chemical (2019) 285, 201–208. <https://doi.org/10.1016/j.snb.2019.01.057> (2019).
3. Chen, P., Peng, Y., He, M., Yan, X., Zhang, Y., & Liu, Y., Sensitive electrochemical detection of creatinine at disposable screen-printed carbon electrode mixed with ferrocenemethanol. Int. J. Electrochem. Sci., 8, 8931 – 8939 (2013).
4. Serafín, V., Hernández, P., Agüí, L., Yáñez-Sedeño, P., & Pingarrón, J. M., Electrochemical biosensor for creatinine based on the immobilization of creatininase, creatinase and sarcosine oxidase onto a ferrocene/horseradish peroxidase/gold nanoparticles/multi-walled carbon nanotubes/Teflon composite electrode. *Electrochimica Acta*, *97*, 175–183. <https://doi.org/10.1016/j.electacta.2013.03.005> (2013) .
